# Treatment costs of long-term invasive meningococcal disease sequelae: A literature review and Delphi study in Brazil

**DOI:** 10.1016/j.bjid.2025.104514

**Published:** 2025-02-19

**Authors:** Noemia Teixeira de Siqueira Filha, Fanny Cortes, Meline Kron, Maira Galdino da Rocha Pitta, Fernando Zanghelini, Bruna de Veras, Tatiane Almeida Menezes, Ana Medina, Lessandra Michelin, Thatiana Pinto

**Affiliations:** a Department of Health Sciences, University of York, York, UK; b Instituto de Avaliação de Tecnologias em Saúde, Porto Alegre, RS, Brazil; c Universidade de Guarulhos, Guarulhos, SP, Brazil; d Universidade Federal de Pernambuco, Recife, PE, Brazil; e GSK, Rio de Janeiro, RJ, Brazil; f GSK, Wavre, Belgium

**Keywords:** Invasive meningococcal disease, Sequelae, Medical resource use, Costs, Brazil

## Abstract

This study describes and estimates the social and economic impact of Invasive Meningococcal Disease (IMD) sequelae globally and in Brazil. An integrative review was conducted to identify IMD sequelae costs estimates worldwide. The evidence identified supported the development of a Delphi survey to estimate medical Resource Use (RU) and caregiver productivity loss during the first Year (Y1) of IMD and the Subsequent Year (SY) in Brazil. Treatment costs of long-term IMD sequelae were estimated through microcosting approach using Brazilian cost reference tables and taking into account the Delphi survey estimates. The review included eight studies from high-income countries. Mean costs of IMD sequelae in high-income countries varied substantially by type of sequelae in Y1 (hearing loss $14,511; amputation $144,087), type of care over a lifetime horizon (outpatient $28,498; inpatient $67,038), and medical procedure over a lifetime horizon (shunt revision $22,794; prosthesis $508,735). The Delphi survey indicated that medical RU was significantly higher in Y1 versus SY. Resource use was highest for patients with multiple limb amputations. In addition, the highest number of outpatient visits (32) were required for patients with skin scars; speech therapy (72) for hearing loss; and the most psychologist sessions (116) for mental health disorders in Y1. Similarly, estimated treatment costs were highest for patients with multiple limb amputations ($4,139.70 in Y1 and $1,874.39 for SY), followed by single limb amputation ($2,803.24 in Y1 and $902.73 for SY) and skin scarring ($2,307.69 in Y1 and $816.19 for SY). All sequelae resulted in multiple workdays lost for caregivers, ranging from 33 (skin scarring) to 85 (multiple limbs amputation) during the first year of treatment. This study informs decision-makers on the healthcare, social and educational services, and social protection needs of patients with long-term sequelae in Brazil and globally.

## Highlights

• Significant costs varying by sequelae type, care level, and medical procedures.
• Delphi survey in Brazil: consensus on high resource use for IMD sequelae, especially in first year.
• Highest costs for amputations with hospitalization, medical care, and therapy.
• Findings aid in understanding patient & family needs, from healthcare to income loss.

## Graphical abstract

**Figure:**
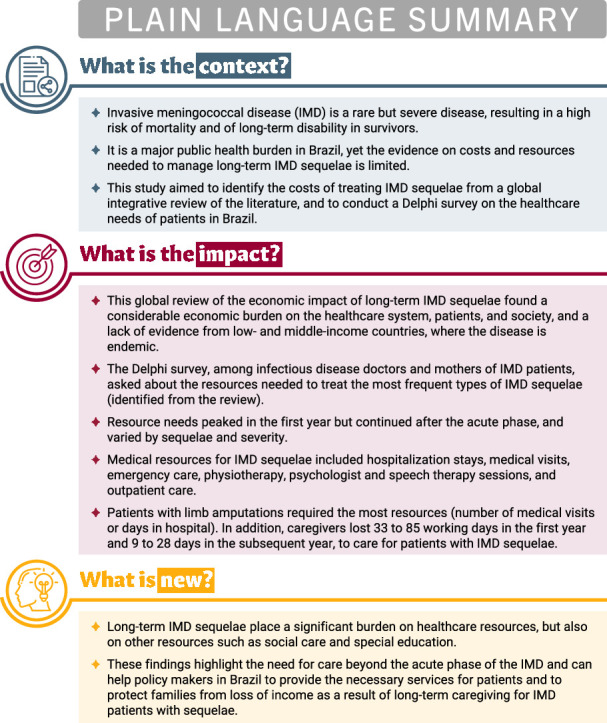

## Introduction

Invasive Meningococcal Disease (IMD), caused by the bacterium *Neisseria meningitidis*, is an uncommon yet severe and life-threatening disease that progresses rapidly and can result in serious and long-term sequelae in survivors. It is the main cause of meningitis and septicemia in children and young adults in Brazil, and is a major public health burden.^1^

IMD is endemic in Brazil, with periods of hyper-endemicity.^1^^,^^2^ Disease incidence has an unpredictable cyclical pattern, varying over time and across regions of Brazil, with the highest rates in infants.^3^ Of the six serogroups that cause most disease, serogroup B and C are now predominant in Brazil^1^ and cause most recent outbreaks.^4^, ^5^, ^6^ Case Fatality Rates (CFR) vary annually, as well as by age group and serogroup. In an observational study using Brazilian surveillance data collected over the 2005‒2018 period, estimated CFRs for meningococcal disease in the overall population was approximately 21 % with no notable differences in any particular year or period.^7^ The CFRs were higher in infants < 1-year, with a mean CFR across 2005–2018 of 23.4 %. The Brazilian Ministry of Health reported a CFR of 24.1 % for serogroup W, 19.2 % for serogroup C, and 17.7 % for serogroup B in 2015.^6^ In a recent outbreak reported in Brazil, the mortality rate for children < 1-year of age was 23.32/100,000 inhabitants and the CFR in this age group reached 75.0 % (for all serogroups).^6^ Most reported cases were identified as serogroup B (72.7 %) and other cases were non-serogrouped (27.3 %).^6^

Studies have estimated that around 20 %–40 % of survivors of the acute phase of IMD will suffer from one or more sequelae.^8^ A systematic review identified a broad range of 30 different physical and neurological sequelae and 14 psychological and behavioral sequelae resulting from IMD. The most important physical and neurological sequelae, with important quality of life and economic consequences, were renal condition, hearing loss, communication disorders, motor deficits, skin scarring, epilepsy, amputations, mental retardation and blindness. For psychological and behavioral sequelae, the most important were anxiety, depression and attention deficit and hyperactivity disorder.^8^ Many of these sequelae were still present in long-term follow-up studies,^8^ highlighting the lifelong burden of disease for IMD survivors and their families and caregivers.^9^

Although some studies outside Latin America have reported the long-term economic burden associated with IMD, few peer-reviewed studies have estimated the costs associated with IMD in Brazil, and these mainly focused on the healthcare system perspective.^4^^,^^10^, ^11^, ^12^ Furthermore, the medical costs of long-term sequelae are significant for healthcare systems and can have an important impact on patients’ families, particularly in low- and middle-income countries. In addition to causing productivity and income losses in caregivers who may give up employment to care for a child with long-term disability, patients with sequelae may require special education for learning disabilities and their long-term work prospects may be affected, they may need rehabilitation and long-term care, and social and financial aid.^13^ Many economic studies do not account for the considerable burden of long-term sequelae on survivors and their families.^8^

A recent systematic review on the global costs of IMD and sequelae reported around 40 % of IMD patients developed sequelae, and healthcare costs were almost twice as high in patients with sequelae versus without. The review included only studies with primary data collection, thus did not identify non-medical and indirect costs of IMD (e.g., productivity losses) and did not report specific costs of sequelae. In addition, most studies only followed patients until discharge, so long-term rehabilitation and care costs were not identified. There were no studies from Brazil.^14^

While the social and economic costs of disabling long-term IMD sequelae are poorly known, the direct impact they have on patients and the indirect consequences for caregivers, families and society are believed to be substantial. However, there is no consensus on the costs and medical resource use needed for treating long-term IMD sequelae in Brazil. The objectives of this study were 1) To identify evidence on the economic burden of long-term IMD sequelae from an integrative review with a broad scope, and 2) To conduct a Delphi survey, informed by the evidence identified, to reach consensus on the medical resource use and associated costs needed to treat long-term IMD sequelae in Brazil.

## Material and methods

An integrative review with a broad scope was conducted to gather evidence to inform the Delphi survey in Brazil.

### Integrative review

An integrative review was conducted (protocol CRD42022299697 registered in PROSPERO platform)^15^ to assess the global evidence on the costs of long-term IMD sequelae, from a payer, patient and societal perspective. The Medline, Embase, Cochrane Library and Lilacs databases were searched on October 20th, 2021 (Supplementary Material available on the Cambridge Core website) (no date restrictions nor filters for country were applied), for studies in English, Portuguese or Spanish, combining search terms relating to meningococcal disease and economic costs. The Rayyan web tool was used for reference management and duplicate removal. Screening of titles and abstracts and full-text articles was conducted by two researchers independently, with any discordances discussed with a third researcher (NTSF or MG) to achieve consensus.

Cost-of-illness studies or economic evaluations in any country addressing the economic impact of long-term sequelae in IMD cases of any age were included. Costs of interest included direct medical costs (e.g., medical resource use and patients’ out-of-pocket expenses for them); direct non-medical costs (e.g., patients’ out-of-pocket expenses for transportation, accommodation and other non-medical resources); indirect costs (e.g., time, income and productivity losses); special education and social care costs; and costs relating to specific sequelae. Studies were excluded if they assessed preventive measures, outbreaks, or diagnosis and treatment without presenting sequelae cost data. Systematic reviews, expert opinion reviews, conference abstracts, editorials, letters, comments, and study protocols were also excluded. Systematic reviews were only considered as a source to identify additional primary studies.

Data were extracted, by two researchers (JM and MK) independently and using the Covidence web tool, on study characteristics (i.e., country, study design, data source, currency, time horizon, study perspective, sample size, age group, and type of sequelae) and outcomes of interest (i.e., direct medical, non-medical and indirect costs, education and social care costs, sequelae-specific costs, cost of medical resources, procedures, services, and length of stay).

The quality of included studies was analyzed using the revised Consolidated Health Economic Evaluation Reporting Standards (CHEERS) checklist.^16^

The costs of IMD sequelae are presented by country, time horizon and cost category (e.g., healthcare, social care, education, indirect costs) with a breakdown by type of cost within each category. Costs relating to specific sequelae are presented separately and categorized by physical, neurological and psychological sequelae.

Cost estimates were converted to 2021 International dollars (I$) using the national gross domestic product deflators and the implied purchasing power parity conversion rates from the International Monetary Fund. Currency conversion rates and inflation adjustments were applied using consumer price indexes.^17^^,^^18^ Studies that did not indicate the year of cost analysis were assumed to have the same base year as the year of publication.

### Delphi survey

A Delphi survey, with two rounds, was conducted to estimate the use of medical and non-medical resources for treating long-term IMD sequelae in Brazil. This type of structured group communication process is used in health sciences to address data gaps, and can provide high levels of evidence when other approaches cannot be used.^19^

Participants (*n* = 33) from Brazil (including infectious disease experts from Brazil's public and/or private health system and parents of IMD patients) were invited to participate based on their experience with the treatment of IMD and familiarity with IMD sequelae.

A questionnaire was designed based on the integrative review findings to enquire about the use of medical resources for the seven most common types of sequelae identified from the review i.e., single limb amputation, multiple limb amputations, skin scarring, epilepsy/seizures, hearing loss/deafness, mental retardation/low IQ (Intelligence Quotient), and mental health disorder/anxiety/depression. The questionnaire was based on a cohort of Brazilian patients with a confirmed long-term IMD sequelae diagnosis and treated in the SUS and/or private health system. Participants were asked to provide information on medical resource use (e.g., medical visits, specialists, hospital days, rehabilitation, home adaptations) in the first Year (Y1) and in the Subsequent Year (SY) by sequela. In addition, participants were asked about indirect costs (e.g., need for paid caregiver, workdays lost) by sequela.

A week before the first round, a summary of the integrative review findings was emailed to all panel participants who agreed to be part of the study and provided informed consent. The questionnaire was then sent to all participants to start the first round. Participants were requested to complete and return the questionnaires to the research team within one week. Results (mean, minimum and maximum values for each question) from the first round were pooled and emailed to participants in the second round for their validation, in order to reach consensus on the final mean values to be reported.

A microcosting analysis based on the Delphi survey estimates was performed to evaluate the costs associated with treatment of long-term IMD sequelae from the public health system (*Sistema Único de Saúde*, SUS) SUS perspective. The codes for the procedures and their unit costs were sourced from the Table of Procedures, Drugs, Orthotics, Prosthetics and Special Materials (SIGTAP) from SUS. Cost data were sourced from public Brazilian databases including SIGTAP.^20^ Hospitals accredited by the SUS are paid by the Ministry of Health budget through reimbursement, and only procedures listed in SIGTAP are reimbursed, using a reference price list to calculate a minimum transfer of values from the Ministry of Health budget to hospitals. Although the reference price list was used for the cost estimates, there may still have been an underestimation of the actual hospital expenditure for long-term IMD sequelae. Considering SIGTAP unit costs only represent federal costs, they were adjusted by a correction factor of 2.8 to obtain the total cost. This correction factor was suggested by the Ministry of Health,^21^ and was also applied by the *Comissão Nacional de Incorporação de Tecnologias* (Brazil's National Commission for the Incorporation of Technologies ‒ CONITEC) in a cost-effectiveness analysis of COVID-19 (Coronavirus Disease 2019) vaccines.^22^^,^^23^ All costs were recorded in Brazilian Reals (R$) and then converted to US dollars (US$) based upon the official exchange rate for Jan‒June 2024 (US $1.00 equals R$5.04).

## Results

### Integrative review

**Fig. 1:**
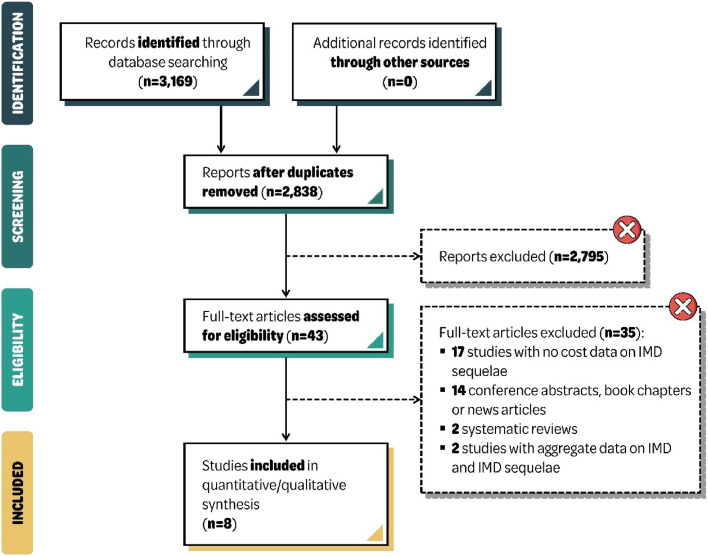
PRISMA flow chart. IMD, Invasive Meningococcal Disease; n, number; PRISMA, Preferred Reporting Items for Systematic reviews and Meta-Analyses.

The search strategy retrieved 2,838 unique records of which eight studies, published from 2011 to 2021, presented costs of IMD sequelae and were included (Fig. 1). All studies reported costs for IMD patients with a range of physical, neurological and psychological sequelae, except one study in the US (United States)^24^ which did not include psychological sequelae. Study designs included cost of illness (*n* = 1)^25^ and cohort studies (*n* = 2),^24^^,^^26^ economic models (*n* = 1),^27^ and studies estimating the costs of hypothetical severe IMD cases presenting with meningitis or septicemia (*n* = 4).^28^, ^29^, ^30^, ^31^ IMD patients of all ages were included. All studies were conducted in high-income countries and there were no studies identified in Latin America. Costs were provided from the payer perspective, patient and/or societal perspectives.

There were two studies in France,^25^^,^^30^ one in Germany,^31^ one in Spain,^29^ one in the United Kingdom,^28^ two in Australia,^26^^,^^27^ and one in the US^24^ (see Table S2 for study details).

Studies in the UK (United Kingdom),^28^ Spain,^29^ and France^30^ estimated the costs associated with the lifetime management of a hypothetical severe case of IMD with sequelae in a young child, presenting either as septicemia or as meningitis. The cases were based on expert and family interviews. In the UK, the lifetime costs of IMD sequelae were assessed from the healthcare payer perspective, including medical, social services, education, and other government costs (e.g., for disability services, lost tax revenue, financial aid).^28^ In Spain, lifetime sequelae costs were estimated from the payer perspective, including medical, education and social care costs.^29^ The French study estimated lifetime costs of IMD sequelae from a payer, societal, and patient and health insurance perspective, including medical costs, education costs and revenue loss for parents.^30^

A cost of illness study in France assessed long-term sequelae costs, up to six years after IMD, using the national claims database (French National Healthcare Data System, [Système National des Données de Santé, SNDS]), in patients of all ages. Costs, from a payer perspective, included medical costs and sick leave and disability compensation.^25^

There were two modelling studies. A model-based costing study in Germany, in a cohort of hypothetical IMD patients of all ages, estimated sequelae costs over 14-years from a societal perspective, including medical and public health response costs, education costs and parental lost productivity costs.^31^ An economic model in Australia estimated the lifetime costs of sequelae in adults with IMD aged 20 or 30 years, from a payer and societal perspective, including medical costs, non-healthcare government subsidies, informal caregiver costs, education costs, home/vehicle modification costs, out-of-pocket costs, and productivity loss and premature death costs.^27^

There were two cohort studies assessing medical costs of IMD sequelae, from a payer perspective, over one year; a cohort study in Australian infants and children included acute hospitalization and readmission costs,^26^ and a US cohort study in IMD patients of all ages included medical costs based on a claims database analysis.^24^

Table 1 presents the breakdown of costs by country, time horizon and cost category, including direct medical costs (e.g., hospital, outpatient), costs for special education needs, social care costs, and indirect lost productivity costs for patients and/or their parent(s). The total costs by country are also presented, which include the costs of treating specific sequelae (presented separately in Table 2 where applicable).

**Table 1:** Breakdown of costs of long-term IMD sequelae by country (International dollars [I$], 2021 prices).

Country source	Horizon	Cost type	Cost (I$)	
			(Y1)	Lifetime (or >Y1°)
Direct medical costs				
Germany^31^	14 Y	a Acute phase (hospital, outpatient, rehabilitation, outbreak management)	NR/NA	17,614
Australia^26^	1 Y	Acute care, readmission	9,490	NR
Australia^26^	1 Y	Acute care, first hospitalization	34,983	NR
Australia^27^	Lifetime	Acute admission	NR	16,859
Spain^29^	Lifetime	Acute care, meningitis	NR	175,669
UK^28^	Lifetime	Acute care, meningitis	NR	238,291
Spain^29^	Lifetime	Acute care, septicemia	NR	216,814
UK^28^	Lifetime	Acute care, septicemia	NR	274,454
US^24^	1 Y	Hospitalization	94,561	NR
France^30^	Lifetime	Hospitalization (Societal), septicemia	NR	87,745
France^30^	Lifetime	Hospitalization (Societal), meningitis	NR	68,710
Germany^31^	14 Y	a Medical cost, sequelae	NR	35,509
France^25^	6 Y	Hospital and community care, multiple sequelae	31,880	NR
France^25^	6 Y	Hospital and community care, multiple sequelae (Y2)	NR	28,427
France^25^	6 Y	Hospital and community care, multiple sequelae (Y5)	NR	12,270
France^25^	6 Y	Hospital and community care, single sequela	20,467	NR
France^25^	6 Y	Hospital and community care, single sequela (Y2)	NR	15,276
France^25^	6 Y	Hospital and community care, single sequela (Y5)	NR	7,119
US^24^	1 Y	Outpatient	16,700	NR
UK^28^	Lifetime	Outpatient, meningitis	NR	35,335
France^30^	Lifetime	Outpatient (Societal), meningitis	NR	28,289
Spain^29^	Lifetime	Outpatient, meningitis	NR	28,435
UK^28^	Lifetime	Outpatient, septicemia	NR	56,936
France^30^	Lifetime	Outpatient (Societal), septicemia	NR	42,512
Spain^29^	Lifetime	Outpatient, septicemia	NR	14,170
Australia^27^	Lifetime	Long-term healthcare cost	NR	12,661
Australia^27^	Lifetime	Long-term disability care	NR	12,059
US^24^	1 Y	Home health/equipment	4,149	NR
US^24^	1 Y	Physician visits	3,507	NR
US^24^	1 Y	Ancillary care	2,544	NR
US^24^	1 Y	Pharmacy	2,237	NR
US^24^	1 Y	Emergency department	1,550	NR
US^24^	1 Y	Laboratory	712	NR
Special education costs				
France^30^	Lifetime	Education, meningitis	NR	1,249,674
Spain^29^	Lifetime	Education, meningitis	NR	240,771
UK^28^	Lifetime	Education, meningitis	NR	9,360
France^30^	Lifetime	Education, septicemia	NR	149,967
Spain^29^	Lifetime	Education, septicemia	NR	47,076
UK^28^	Lifetime	Education, septicemia	NR	8,118
Social care costs				
Spain^29^	Lifetime	Social care, meningitis	51,888	1,431,364°
UK^28^	Lifetime	Social care, meningitis	NR	28,368
Spain^29^	Lifetime	Social care, septicemia	44,626	843,744°
UK^28^	Lifetime	Social care, septicemia	NR	4,175
Indirect costs				
Germany^31^	14 Y	a Productivity loss, patient or parent (Friction cost)	NR	1,783
Germany^31^	14 Y	a Productivity loss, patient or parent (Human capital approach)	NR	129,953
France^30^	Lifetime	Revenue loss, parent, meningitis	NR	238,064
France^30^	Lifetime	Revenue loss, parent, septicemia	NR	115,432
Total costs by country and perspective				
France^30^	Lifetime	Total (Patient/Insurance), meningitis	NR	386,922
		Total (Patient/Insurance), septicemia	NR	152,237
		Total (Payer), meningitis	NR	2,490,100
		Total (Payer), septicemia	NR	997,204
		Total (Societal), meningitis	240,163	2,877,023°
		Total (Societal), septicemia	249,495	1,149,441°
Australia^27^	Lifetime	a Total (Payer)	NR	14,593
		a Total (Societal, Friction cost)	NR	25,188
		a Total (Societal, Human capital approach)	NR	56,707
Spain^29^	Lifetime	Total (Dictionary, #143), meningitis	189,767	532,076°
		Total (Dictionary, #143), septicemia	261,993	951,294°
		Total (Societal), meningitis	NR	2,204,209
		Total (Societal), septicemia	NR	1,842,115
UK^28^	Lifetime	Total (Societal), meningitis	701,095	4,907,644°
		Total (Societal), septicemia	571,271	3,426,176°
US^24^	1 Y	Total (Payer)	125,961	NR

IMD, Invasive Meningococcal Disease; UK, United Kingdom; US, United States; Y, Year; NR, Not Reported.

° Some studies split costs by Year 1 and > Year 1 costs.

a Scholz et al. (2019)^31^ and Wang et al. (2019)^27^ provided some average costs per IMD case including non-sequelae cases.

**Table 2:** Mean costs relating to specific sequela, by country (International dollars [I$], 2021 prices).

Country	Horizon		Costs (I$)	
			Y1	Lifetime (or > Y1)
**Physical sequelae**				
France^25^	5 Y	Amputation	54,018	23,653
Australia^23^	Lifetime	Amputation, digit		36,805
Australia^23^	Lifetime	Amputation, single limb		141,937
Australia^23^	Lifetime	Amputation, multiple limbs		253,519
Australia^23^	Lifetime	Arthritis		6,695
France^25^	5 Y	Motor deficits	19,974	7,421
France^30^	Lifetime	Prosthesis		420,975
UK^22^	Lifetime	Prosthesis		452,974
Spain^21^	Lifetime	Prosthesis		652,225
France^25^	5 Y	Renal disease	25,673	12,895
Australia^23^	Lifetime	Renal failure, chronic		281,316
Australia^23^	Lifetime	Skin grafting		8,969
Spain^21^	Lifetime	Stump revisions, skin grafting		25,310
UK^22^	Lifetime	Stump revisions, skin grafting		38,072
France^25^	5 Y	Skin scarring	44,870	19,262
**Neurological sequelae**				
France^25^	5 Y	Blindness, severe visual impairment	14,146	8,025
Australia^23^	Lifetime	Blindness		44,203
Australia^23^	Lifetime	Brain injuries		25,402
Australia^23^	Lifetime	Chronic migraine		11,038
France^25^	5 Y	Epilepsy	19,031	6,223
UK^22^	Lifetime	Epilepsy		6,532
Australia^23^	Lifetime	Epilepsy		80,858
Spain^21^	Lifetime	Epilepsy		99,610
Australia^23^	Lifetime	Hearing loss, adaptation strategies		6,435
Australia^23^	Lifetime	Hearing loss, hearing aid		15,920
Germany^20^	14 Y	Hearing loss		25,509
France^25^	5 Y	Hearing loss, unilateral	10,504	2,065
France^25^	5 Y	Hearing loss, bilateral	35,495	4,900
France^25^	5 Y	Hearing loss, cochlear implant	13,841	2,719
Spain^21^	Lifetime	Hearing loss, cochlear implant		14,349
Australia^23^	Lifetime	Hearing loss, cochlear implant		21,179
UK^22^	Lifetime	Hearing loss, cochlear implant		306,564
France^25^	5 Y	Mental retardation	28,988	29,802
France^25^	5 Y	Neurological deficit, severe	22,129	9,879
Spain^21^	Lifetime	Shunt revision surgery		12,689
France^30^	Lifetime	Shunt revision surgery		23,181
UK^22^	Lifetime	Shunt revision surgery		32,511
France^25^	5 Y	Speech or communication problems	19,917	4,518
Australia^23^	Lifetime	Speech problems, severe		25,402
**Psychological sequelae**				
France^25^	5 Y	Anxiety	11,216	2,215
Australia^23^	Lifetime	Anxiety, generalized disorder		11,038
UK^22^	Lifetime	Behavioral problems		14,643
France^25^	5 Y	Depression	17,753	6,074
Australia^23^	Lifetime	Depression		15,201
France^25^	5 Y	Hyperactivity syndrome	14,955	1,770
Germany^20^	14 Y	Psychological problems		10,816
Spain^21^	Lifetime	Psychological problems		36,283

UK, United Kingdom; Y, Years.

Significant differences were observed for costs of education (range: I$8,118–I$1,249,674) and social care (range: I$4,175–I$843,744), which can vary according to the type and number of sequelae and the type or availability of services. For education, services may include special education needs, learning support assistance, school adaptations and equipment, transport from and to school, and specific services such as speech and language therapy and physiotherapy. For social care, costs may include day-care centers for activities, social care assessments, home visits and reviews.^28^, ^29^, ^30^

Costs associated with specific physical, neurological or psychological sequelae were reported in all countries except the US (Table 2). Costs of managing each sequela differed by study, with the highest costs observed for amputations, prosthesis, and chronic renal failure, among physical sequelae; hearing loss requiring cochlear implant and epilepsy among neurological sequelae; and psychological problems and depression, among psychological sequelae (Table 2). From the payer perspective, mean costs varied substantially by type of sequelae (e.g., I$14,511 for hearing loss versus I$144,087 for amputation), by type of care (e.g., I$28,498 for outpatient care versus I$67,038 for inpatient care), and by medical procedure (e.g., I$22,794 for shunt revision versus I$508,735 for prosthesis).

**Fig. 2:**
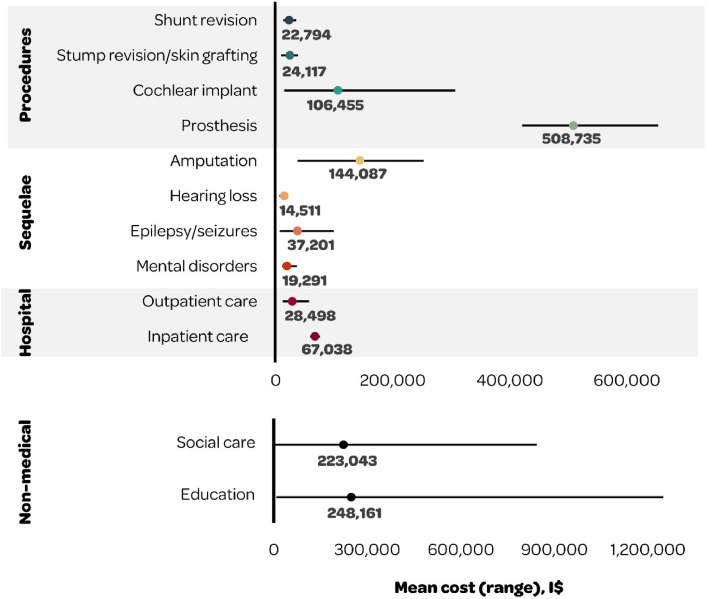
Mean discounted lifetime costs and range (I$) of long-term IMD sequelae. I$, International Dollars; IMD, Invasive Meningococcal Disease.

Mean costs were calculated across studies adopting the provider perspective and applying a lifetime horizon (Fig. 2). The unweighted mean lifetime costs were: I$67,038 (range: I$58,314–75,763)^30^ for inpatient care; I$28,498 (range: I$12,059–56,936) for outpatient care^27^, ^28^, ^29^, ^30^; and for sequelae, they were I$19,291 for mental disorders (range I$11,038 for anxiety to I$36,283 for general psychological problems)^27^, ^28^, ^29^; I$37,201 (range: I$6,532–99,610) for epilepsy/seizures^27^, ^28^, ^29^; I$14,511 for hearing loss (range: I$6,435 for hearing loss requiring adaptation strategies to I$21,179 for cochlear implant)^27^ ; I$144,087 for amputation (range: I$36,805 for single amputation to I$252,519 for multiple amputations).^27^ The unweighted mean lifetime costs of medical procedures^27^, ^28^, ^29^, ^30^ were I$508,735 (range: I$420,975–653,255) for prosthesis; I$106,455 (range I$14,349–306,564) for cochlear implant; I$24,117 (range I$8,969–38,072) for stump revision and skin grafting; and I$22,794 (range: I$12,689–35,511) for shunt revision. The mean discounted lifetime cost of education was I$248,161 (range: I$8,118–1,249,674) and of social care, I$223,043 (range: I$4,175–843,744).^28^, ^29^, ^30^

### Delphi panel

The previously described evidence was used to support the development of a Delphi questionnaire aimed to estimate medical Resource Use (RU) and caregiver productivity loss during the First Year (Y1) and the Subsequent Year (SY) for the most frequently reported sequelae in the literature (e.g., amputation, skin scars, hearing loss, epilepsy, mental retardation and mental health disorders, Fig. S1). Of the 33 participants invited to participate in the Delphi survey, five confirmed participation and provided informed consent, including three infectious disease doctors and two mothers of IMD patients. The survey group included individuals with diverse backgrounds and perspectives i.e., healthcare providers versus patients’ parents, public versus private sector, from different regions of Brazil (i.e., São Paulo in the Southeast, Pernambuco in the Northeast, Paraná in the South, Minas Gerais in the Southeast, and Rio de Janeiro in the Southeast).

All participants completed and returned the questionnaire in Round 1 and provided validation of the pooled results in Round 2. The Delphi survey reached a consensus on the mean, minimum and maximum values for most resources used. However, one participant did not answer all the questions and disagreed with the values reported for the number of physiotherapy sessions (i.e., participant reported 8 vs. 6 from the other participants) and the average number of working days lost (i.e., participant reported no value for SY vs. 28 days from the other participants). Both questions related to multiple limb amputations after the first year.

Additional comments provided by the participants highlighted that severity can have an important impact on the burden of IMD sequelae, including the need for rehabilitation or consultations; and the presence of multiple sequelae can affect burden and costs significantly. A case story by one participant highlighted the nature of long-term healthcare resource use and indirect costs (e.g., four months in the intensive care unit, various procedures and treatments including tracheostomy for seven months, need for home oxygen, prosthesis and use of orthoses due to amputations which required changes with growth, the need for a caregiver, and the need for parental caregiving as home care was only provided for eight months).

**Fig. 3:**
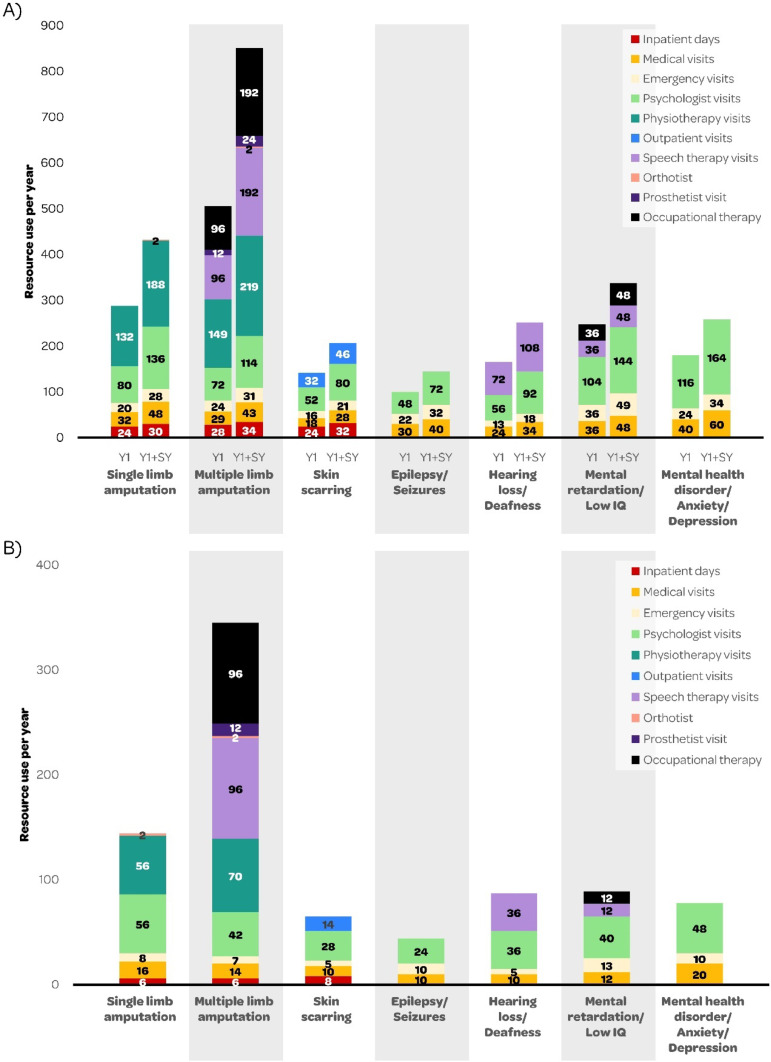
Mean medical resource use (days or visits) by IMD sequela in (A) Year 1 (Y1) and Y1 + Subsequent Year (SY) and (B) subsequent year ‒ results from the Delphi survey for Brazil. IMD, Invasive Meningococcal Disease; IQ, Intelligence Quotient.

From the results of the Delphi survey, Fig. 3 presents the mean medical resource use needed to treat the seven sequelae, in the first Year (Y1) and the Subsequent Year (SY) of IMD (see Supplementary File for Delphi results by round).

The use of medical resources was significantly higher during Y1 compared with SY for all sequelae. Patients with single limb and multiple limb amputations had the highest overall medical resource use. All cases with a sequela required medical doctor visits (18‒40 in Y1 vs. 10‒20 SY), emergency department visits (13‒36 in Y1 vs. 5‒13 SY) and psychologist visits (48‒116 in Y1 vs. 24‒56 SY). Patients with amputations and skin scarring also required many inpatient hospital days (24‒28 in Y1 vs. 6‒8 SY). Patients with single or multiple limb amputations required physiotherapy visits (132‒149 in Y1 vs. 56‒70 SY) and orthotist visits (2 SY), and those with multiple limb amputation required 12 prosthetist visits (in Y1 and SY). There were 96 occupational therapist visits (in Y1 and SY) for patients with multiple limb amputations, and also for patients with mental retardation (36 in Y1 vs. 12 SY). Patients with skin scarring required outpatient visits for dressing (32 in Y1 vs. 14 SY). Patients with hearing loss or deafness required speech therapy visits (72 in Y1 vs. 36 SY) (Fig. 3).

**Fig. 4:**
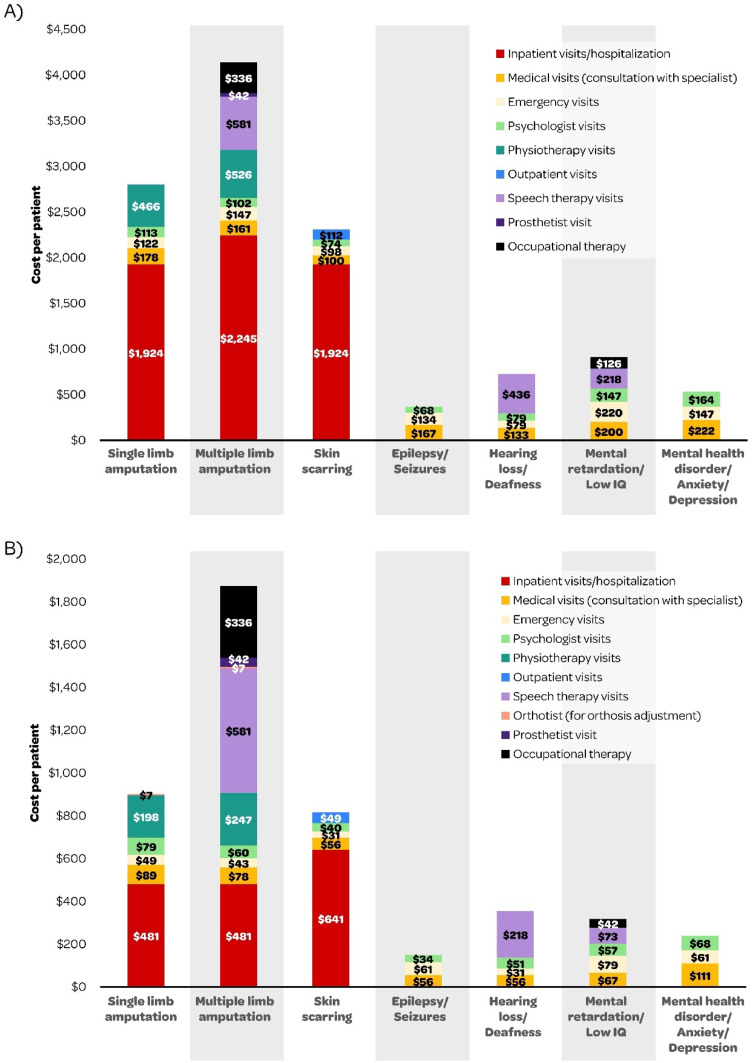
Total cost per patient (USD) by IMD sequela in (A) Year 1 (Y1) and (B) Subsequent Year (SY) ‒ results from the microcosting approach for Brazil. IMD, Invasive Meningococcal Disease; USD, US Dollar.

The estimated per-patient costs of medical resource use to treat long-term IMD sequelae in the first and subsequent years of IMD are presented in Fig. 4. For amputation of a single limb, we estimated a cost of $2,803.24 and $902.73 for Y1 and SY, respectively. These costs were almost doubled ($4,139.70 in Y1 and $1,874.39 in SY) for multiple limb amputation. Costs for treatment of skin scarring sequelae were $2,307.69 in Y1 and $816.19 in SY. For IMD-associated seizures, we estimated that the per-patient cost was $369.11 in Y1 and $150.67 for SY, excluding the anticonvulsant therapy (which are mainly out of pocket expenses and reported by one of the respondents in the Delphi panel). For hearing loss, Y1 and SY costs were estimated at $728.11 and $355.11, respectively. It is noteworthy that hearing aid costs are not included in these estimates. Medical resource use costs were also estimated for mental retardation ($911.33 in Y1 and $317.44 for SY) and IMD-associated mental health disorders ($533.22 in Y1 and $240.22 for SY).

**Fig. 5:**
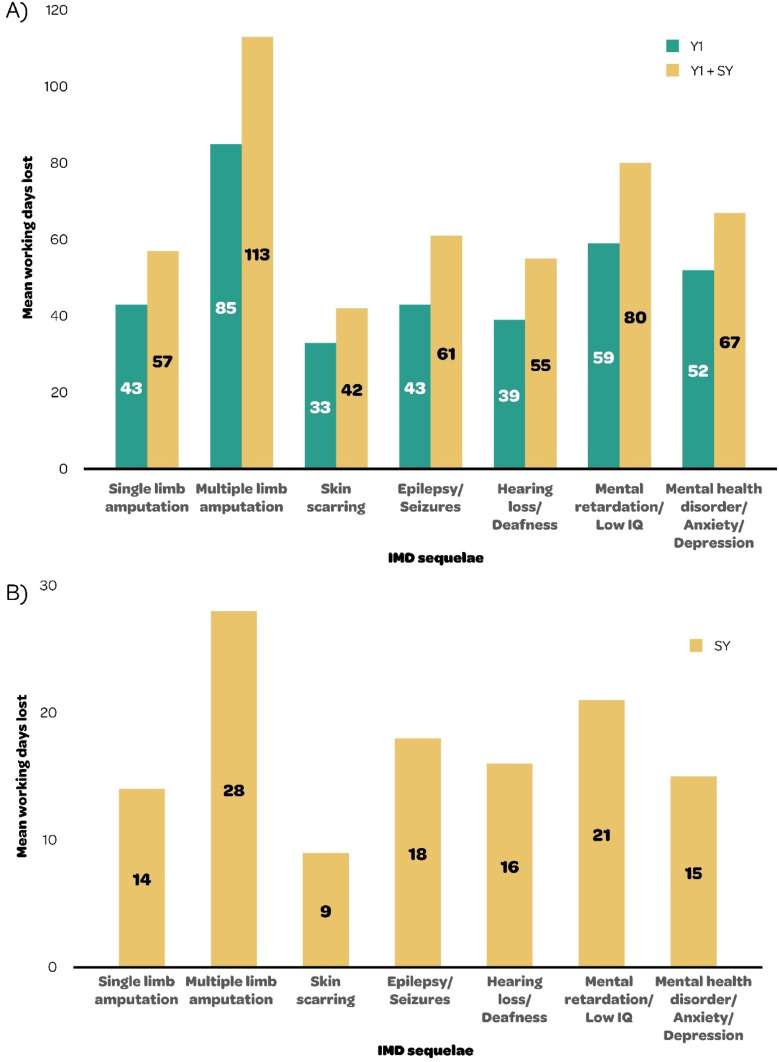
Mean number of working days lost by sequela, in (A) Year 1 (Y1) and Y1 + Subsequent Year (SY) and (B) subsequent year ‒ results from the Delphi survey for Brazil. IMD, Invasive Meningococcal Disease; IQ, Intelligence Quotient; Y, Year.

Fig. 5 presents the mean number of working days lost for the treatment of the seven long-term IMD sequelae, in Y1 and Y1 + SY.

The Delphi survey shows that all sequelae were associated with multiple working days lost, ranging from 33 for skin scarring to 85 for multiple limb amputations in Y1, and from 9 for skin scarring to 28 for multiple limb amputations SY. The mean number of working days lost was considerably higher in Y1 versus SY for all sequelae, representing an important indirect cost driver (Fig. 5). In addition, participants reported needing a paid caregiver for most of the sequelae in Y1 and SY.

## Discussion

This study used an integrative review to identify the costs of long-term IMD sequelae from the payer, patient and societal perspectives. The findings informed a Delphi survey achieving a consensus from experts on the medical resources needed to treat specific IMD sequelae and their cost in Brazil, as well as the associated working days lost.

The review captured evidence from only high-income countries, on long-term direct medical costs, education and social care costs, and lost productivity costs associated with a range of physical, neurological and psychological IMD sequelae. There is a lack of evidence for Brazil, South America and other developing/emerging upper-middle income countries. The Delphi survey, among five medical experts and parents of IMD patients with sequelae, was the first study to provide information for Brazil on medical resource use and workdays lost for IMD sequelae. The findings highlight the significant resources needed, especially in the first year following IMD, for physical sequelae (e.g., limb amputations, skin scarring), neurological sequelae (e.g., epilepsy, hearing loss, mental retardation) and psychological sequelae (e.g., anxiety, depression). Patients with multiple limb amputations had the highest resource use (e.g., 28 inpatient days, 24 emergency visits, 29 doctor visits, 12 prosthetist visits, 72 psychologist visits, 96 speech therapy and occupational therapy visits, and 149 physiotherapy sessions in the first year alone), findings aligned with the integrative review. In the subsequent year, there was a decline in the number of inpatient days (6), medical visits (14), emergency visits (7), psychologist visits (42) and physiotherapy visits (70), other medical resource use remained high e.g., speech therapy (96) and occupational therapy (96). Participants commented on patients’ needs for long-term rehabilitation, and the presence of multiple sequelae. In one case story, the participant described the continuous need for changes to prostheses as the patient grew. These examples highlight the likely lifelong need for medical and other resources, which may not be fully captured in the Delphi survey. The microcosting approach allowed for an estimation of per-patient treatment costs, showing the substantial economic burden associated with the selected IMD sequelae. It is noteworthy that purchase of provisional and permanent orthosis as well as hearing aid costs and anticonvulsant use (for IMD-associated seizures) are not included in these estimates. In addition to the high healthcare burden, there was an important indirect cost driven by workdays lost; as caregivers of patients with IMD sequelae can expect to lose 33 to 85 working days in the first year due to sequelae.

Treatment cost estimates add to the body of evidence related to the economic burden of IMD in Brazil that has been mainly assessed from the healthcare perspective.^4^^,^^10^, ^11^, ^12^ In a study focusing on outbreak costs (i.e., disease surveillance and outbreak management), total costs have been estimated as high as three times the gross domestic product per capita.^4^ Another study estimated the direct hospital costs (2008‒2018) of several vaccine-preventable diseases; for IMD, over 24,000 hospitalizations occurred during the study period (almost 245,000 hospital days) costing the health system R$ 47,156,734.49.^11^ A cost of illness study (only available as a conference abstract) estimated the direct medical costs (from the Unified Health System [*Sistema Único de Saúde*, SUS]) of IMD in 2,651 infants with IMD from 2007 to 2015, and the costs of their sequelae until the age of 18 years. The study assumed 23.9 % of cases had at least one sequela, the most common being deafness (28 %), epilepsy (28 %), amputation (9 %) and skin necrosis (9 %). The acute phase costs for diagnosis, inpatient care and chemoprophylaxis of contacts amounted to US$1.1 million (2016USD); and sequelae treatment costs were estimated at US$2.1 million, of which the majority were for deafness (66 %), followed by amputation (20 %), epilepsy (12 %) and skin necrosis (2 %).^12^ A literature review, published in 2013 and only available as a conference abstract, identified the cost burden of IMD in children in terms of direct costs (e.g., medical visits, hospitalization, laboratory tests, imaging studies, medication, and transportation to health services), and indirect costs from caregiver productivity losses. The direct costs of an IMD episode ranged from R$1,000–1,500 (2006 Brazilian Reals) in children under nine years, and sequelae costs were highest for neurological impairment (R$8,053 and R$19,154 including indirect costs).^10^ In a cost-effectiveness analysis assessing the impact of meningococcal vaccination in Brazil, authors estimated the input parameters for costs (direct and indirect) of treatment for IMD sequelae to range from R$5,569 (necrosis and amputation) to R$48,259 (neurological and hearing impairments).^32^ Variations in cost estimates were observed across the literature, depending on the type of care, treatments, and time-horizon taken into account. As highlighted by Itria and colleagues, input parameters for treatment costs of sequelae may substantially impact the cost-effectiveness assessment of vaccination.^32^

The integrative review was able to capture a broader range of study designs than traditional systematic reviews, including bottom-up costing studies for severe IMD cases with long-term disabilities, that were defined from expert interviews. These data provide new and important insights into the long-term needs of IMD patients and their families, and the significant healthcare, patient and societal costs of the disease. Key strengths of the Delphi survey were the inclusion of healthcare providers from public and private health sectors in Brazil, and parents of IMD patients providing a family perspective and details from a case study. The methodological approach, using anonymous participation and allowing freedom of expression, made it possible to avoid conflicts of interest within the group and to minimize dominance of researchers from any region that could influence the results.

No sequelae costing data were identified from low- and middle-income countries or Latin America, where the financial burden on patients and families may be even more severe due to precarious healthcare systems, and difficult to access or unavailable long-term and specialized healthcare services. In these circumstances, patients and caregivers are more likely to incur severe economic loss due to high out-of-pocket expenditures, income loss and reduced productivity.^13^ Impoverishment as a result of catastrophic health expenditure^33^ was not considered by any of the studies included in this review, but may well be applicable for IMD cases in developing countries. Furthermore, vulnerable people in the lowest socio-economic strata are at greater risk of infection, for example, from living in overcrowded housing. The risk and economic burden of IMD and its long-term sequelae may also be increased due to multimorbidity, such as HIV/AIDS (human immunodeficiency virus/acquired immunodeficiency syndrome) and smoking, which are also more prevalent in these countries.^34^

This study also has some limitations. The integrative review included cost estimates from severe case studies (i.e., studies reporting costs for two severe patients) which may not be generalized to all IMD patients. While healthcare costs were provided for specific sequelae in some studies, the indirect, education and social costs were not broken down by sequela. Heterogeneity in the types and severity of sequelae, the cost components included, and the study designs and methodologies made comparisons difficult. Sequela costs are likely to vary by severity, however, this level of detail was not available. Pooling data across studies was difficult, therefore, due to heterogeneity across countries and settings, differences in time horizons used, and lack of data on the severity of sequela, which is a major driver of resource use and costs. Low recruitment in the Delphi survey, with no participants from the North and Midwest regions of Brazil, also affected the generalizability of the findings, given the regional and cultural diversity in Brazil. In addition, as IMD can have a wide range of sequelae which can vary in severity across patients, the findings cannot be generalized across the IMD population, due to the low number of participants in the Delphi survey. More research is needed to obtain more granular data across a range of IMD patients from multiple regions of Brazil.

While the integrative review and Delphi survey confirmed that long-term IMD sequelae impose a severe economic burden on patients and the healthcare system, evidence gaps remain concerning patient out-of-pocket costs and the impact specific to low and middle-income countries. Future studies may address these gaps by adopting standardized costing approaches in Latin American countries to provide more accurate evidence for the region.

This study provides valuable information on the types of medical resources and non-health services needed to support IMD patients with long-term sequelae and can help inform policy makers about the need to finance and improve access to such services. The study also shows the high healthcare burden and budget impact of long-term sequelae. Results indicate that indirect costs from caregiver lost productivity contribute to the economic burden, and public policies and social protection mechanisms may be needed to protect families from financial hardship as a consequence of IMD sequelae.

## Authors’ contributions

Concept and design: Thatiana Pinto.

Acquisition of data: Noemia Teixeira de Siqueira Filha, Fanny Cortes, Meline Kron, Maira Galdino da Rocha Pitta, Fernando Zanghelini.

Analysis and interpretation of data: Noemia Teixeira de Siqueira Filha, Fanny Cortes, Meline Kron, Maira Galdino da Rocha Pitta, Fernando Zanghelini, Bruna de Veras, Tatiane Almeida Menezes, Ana Medina, Lessandra Michelin, Thatiana Pinto.

Drafting of manuscript: Noemia Teixeira de Siqueira Filha, Fanny Cortes, Meline Kron, Fernando Zanghelini, Thatiana Pinto, Bruna de Veras.

Critical revision of the paper for important intellectual content: Noemia Teixeira de Siqueira Filha, Fanny Cortes, Meline Kron, Maira Galdino da Rocha Pitta, Fernando Zanghelini, Bruna de Veras, Tatiane Almeida Menezes, Ana Medina, Lessandra Michelin, Thatiana Pinto.

Obtaining funding: Noemia Teixeira de Siqueira Filha, Fanny Cortes, Meline Kron, Maira Galdino da Rocha Pitta, Fernando Zanghelini, Bruna de Veras, Tatiane Almeida Menezes, Ana Medina, Lessandra Michelin, Thatiana Pinto.

Administrative, technical, or logistic support: Noemia Teixeira de Siqueira Filha, Fanny Cortes, Meline Kron, Maira Galdino da Rocha Pitta, Fernando Zanghelini, Bruna de Veras, Tatiane Almeida Menezes, Ana Medina, Lessandra Michelin, Thatiana Pinto.

Supervision: Thatiana Pinto.

## Financial support

GSK funded this study (VEO-000045). GSK was involved in all stages of study conduct, including analysis of the data. GSK also took in charge all costs associated with the development and publication of this manuscript.

## Conflicts of interest

Ana Medina, Bruna de Veras, Lessandra Michelin and Thatiana Pinto are employed by GSK. Thatiana Pinto holds financial equities in GSK. Maira Galdino da Rocha Pitta, Noemia Teixeira de Siqueira Filha and Tatiane Almeida Menezes received funding from GSK for the submitted work. These authors declare no other financial and non-financial relationships and activities. Fanny Cortes, Fernando Zanghelini and Meline Kron declare no financial and non-financial relationships and activities and no conflicts of interest.

## Appendix Supplementary materials

Image, application 1
