# Supplementary material for: Treatment costs of long-term invasive meningococcal disease sequelae: A literature review and Delphi study in Brazil
Source: Braz J Infect Dis. 2025 Feb 19;29(2):104514. doi: 10.1016/j.bjid.2025.104514 (PMC11889580; doi:10.1016/j.bjid.2025.104514)
Supplement: Supplementary file 1 [file mmc1.pdf]

Supplementary material

Social and economic impact of invasive meningococcal disease sequelae – A global integrative review and Delphi study in Brazil

Table S1. Search strategy integrative review

#	Query	Results from 21 October 2021
1	Economics/	27,377
2	"costs and cost analysis"/	50,054
3	Cost allocation/	2,010
4	Cost-benefit analysis/	86,786
5	Cost control/	21,613
6	Cost savings/	12,388
7	Cost of illness/	29,720
8	Cost sharing/	2,632
9	"deductibles and coinsurance"/	1,793
10	Medical savings accounts/	543
11	Health care costs/	42,247
12	Direct service costs/	1,207
13	Drug costs/	16,842
14	Employer health costs/	1,095
15	Hospital costs/	11,644
16	Health expenditures/	22,079
17	Capital expenditures/	1,998
18	Value of life/	5,766
19	exp economics, hospital/	25,348
20	exp economics, medical/	14,296
21	Economics, nursing/	4,006
22	Economics, pharmaceutical/	3,024
23	exp "fees and charges"/	30,918
24	exp budgets/	13,909
25	(low adj cost).mp.	69,398
26	(high adj cost).mp.	16,651
27	(health?care adj cost$).mp.	13,711
28	(fiscal or funding or financial or finance).tw.	166,959
29	(cost adj estimate$).mp.	2,490
30	(cost adj variable).mp.	47
31	(unit adj cost$).mp.	2,783
32	(economic$ or pharmacoeconomic$ or price$ or pricing).tw.	344,088
33	or/1-32	812,728
34	exp Neisseria meningitidis/ or exp Meningitis, Meningococcal/ or exp Meningococcal Infections/ or Invasive meningococcal disease.mp.	16,073
35	33 and 34	341

Table S2. Study characteristics and results (costs in International dollars [I$], 2021 prices).

Study	Study design and population	Sequelae	Type of cost/impact and outcomes	Results
Weil-Olivier 2021[1]<br><br>France SNDS database	Cost of illness alongside case-control (2012–2017)<br>**N**=3,530 cases<br>Infants, children, adolescents and adults (≤ 25 years, 25 to 49 years, ≥50 years)<br><br>Societal perspective<br><br>Time horizon: 12 months and 5 years	***Physical sequelae:*** motor deficits, renal disease, skin scarring<br>***Neurological sequelae:*** Hearing loss, epilepsy, mental retardation, neurological deficit, visual impairment/blindness, speech or communication problems<br>***Psychological sequelae:*** anxiety, depression, hyperactivity syndrome,	<u>Costs:</u> direct medical costs (hospitalization or community care, out-of-pocket expenses), indirect cost (sick leave and invalidity pension, from the payer perspective).<br>Cost of hospitalization (general hospital stays/rehospitalizations, rehabilitation stay, psychiatric unit, intensive care unit, home care): remuneration of physicians and paramedical staff, medications and medical devices delivered in hospital and routine tests, specific procedures (e.g., dialysis).<br>Cost of community care: physician visits, visits by nurses and other paramedical professionals, delivery of medication and medical devices in pharmacies, clinical laboratory tests and medical transportation.<br>Indirect costs: sick leave and invalidity pensions (payer perspective)<br><br><u>Outcome</u>: First year and 5 years per capita costs (by year of follow-up), costs of management of long-term sequelae (one or more), annual	*Mean cost of index case with single sequelae:* $20,467<br>*Mean cost of index case with multiple sequelae:* $31,880<br>*Mean per capita costs in the year following index hospitalization:*<br>One sequela: $15,276<br>Multiple sequelae: $28,427<br>*Mean per capita costs (5th year after the index hospitalization):*<br>One sequela: $7,119<br>Multiple sequelae: $12,270<br><br>*Mean annual per capita costs of management of long-term sequelae*<br>Amputation: 1st year $54,018; subsequent year $23,653<br>Anxiety: 1st year $11,216; subsequent year $2,215<br>Bilateral hearing loss: 1st year $35,495; subsequent year $4,900<br>Depression: 1st year $17,753; subsequent year $6,074<br>Epilepsy: 1st year $19,031; subsequent year $6,223<br>Hearing loss requiring cochlear implant: 1st year $13,841; subsequent year $2,719<br>Hyperactivity syndrome: 1st year $14,955; subsequent year: $1,770<br>Mental retardation: 1st year $28,988; subsequent year $29,802

			per capita costs, mean duration of the index hospitalization	Motor deficits: 1st year $19,974; subsequent year $7,421<br>Renal disease: 1st year $25,673; subsequent year $12,895<br>Severe neurological deficit: 1st year $22,129; subsequent year $9,879<br>Severe visual impairment/blindness: 1st year $14,146; subsequent year $8,025<br>Skin scarring: 1st year $44,870; subsequent year $19,262<br>Speech or communication problems: 1st year $19,917; subsequent year $4,518<br>Unilateral hearing loss: 1st year $10,504; subsequent year $2,065
Benard 2016[2]<br><br>France	Two hypothetical cases (based on literature, National Health Insurance (NHI) data, interviews and case studies)<br>Child aged 3 with meningitis, severe neurological sequelae<br>Child aged 6 with purpura fulminans, amputation of both legs below the knee<br>Societal, provider, and patient and health insurance perspectives<br><br>Time horizon: 12 months and lifetime (77 years,	***Patient with septicemia sequelae***<br>***Physical sequelae:*** Amputation, post-amputation complications, dermatological damage, chronic kidney disease<br>***Neurological sequelae:*** cognitive deficiencies, complete hemiplegia, lateral homonymous hemianopsia, hydrocephalus with ventriculoperitoneal shunt, neurological sequelae<br>***Psychological sequelae:*** behavioral disorder	Costs: Direct medical costs (consultations, transports, hospitalizations, rehabilitation center, drugs, prostheses, wheelchairs, crutches, pressure garments, medicalized stroller, support corset, cochlear implants, diaper), indirect medical costs (biological tests, blood sampling, public health doctor, personal and educational assistant salaries, chemoprophylaxis, vaccines and drugs, accommodation, car adaptation, pediatric residential unit, full-time residential care), indirect costs (mother's revenue loss, financial aid)	Patient A<br>*Provider perspective, lifetime costs, discounted cost*<br>Ambulatory care: $35,939<br>Hospitalization: $75,763<br>Prosthesis: $420,975<br>Education: $149,967<br>Revenue loss: $32,211<br>Total costs, discounted (undiscounted): $997,204 ($1,513,036)<br>*Patient and health insurance perspective, lifetime costs, discounted cost*<br>Ambulatory care: $6,575<br>Hospitalization: $11,984<br>Parent's revenue loss: $83,211

	patient A; 55 years patient B)		<u>Outcome</u>: Discounted and undiscounted total cost of IMD from symptom onset to patient death. Discounted and undiscounted annual mean cost	Total cost, discounted (undiscounted): $152,237 ($258,829)<br>*Societal perspective, lifetime costs, discounted cost*<br>Ambulatory care: $42,512<br>Hospitalization: $87,745<br>Prosthesis: $420,975<br>Education: $149,967<br>Revenue loss: $115,432<br>Total cost, discounted (undiscounted): $1,149,441 ($2,172,919)<br>Societal 12 months cost: $249,495<br><br><u>Patient B</u><br>*Provider perspective, lifetime costs, discounted cost*<br>Ambulatory care: $16,593<br>Hospitalization: $58,314<br>Shunt revision: $23,181<br>Education: $1,249,674<br>Revenue loss: $43,556<br>Total costs, discounted (undiscounted): $2,490,100 ($5,194,545)<br>*Patient and health insurance perspective, lifetime costs, discounted cost*<br>Ambulatory care: $11,697<br>Hospitalization: $10,396<br>Shunt revision: $184<br>Parent's revenue loss: $194,510

				Total cost, discounted (undiscounted): $386,922 ($668,493)<br>*Societal perspective, lifetime costs, discounted cost*<br>Ambulatory care: $28,289<br>Hospitalization: $68,710<br>Shunt revision: $23,366<br>Education: $1,249,674<br>Revenue loss: $238,064<br>Total cost, discounted (undiscounted): $2,877,023 ($5,862,635)<br>Societal 12 months cost: $240,163
Scholz 2019[3]<br><br>Germany	Model<br>Hypothetical cohort of 343 IMD patients (study period 2001–2016)<br>Statistics from the Federal Statistical Office, expert opinion<br>Ages <1 year, 1–4 years, 5–9 years, 5-year age groups until age 80+<br><br>Societal perspective<br><br>Time horizon: 14 years	***Physical sequelae:*** limb amputation, skin scarring, renal disease,<br>***Neurological sequelae:*** Hearing loss, blindness, neurological disability, seizures/epilepsy<br>***Psychological sequelae:*** attention deficit, anxiety, separation anxiety	<u>Costs</u>: Direct costs (inpatient, outpatient, rehabilitation, special education and long-term care), indirect costs (reduced productivity due to long-term consequences of sequelae in IMD survivors)<br><u>Outcomes</u>: Total cost, cost of sequelae	Mean medical costs of sequelae: $35,509/IMD survivor<br>Mean medical costs psychological impairments: $10,816/IMD survivor<br>Mean medical costs hearing loss: $25,509/IMD survivor<br>Mean indirect (i.e., reduced productivity of patients or parents caring for their child)<br>Friction approach: $1,783<br>Human capital approach: $129,953
Darba 2014[4]<br><br>Spain	Two hypothetical cases (based on interviews, case studies, national or local tariff lists or catalogues, Spanish Medication database, published data and	***Physical sequelae:*** Amputation<br>***Neurological sequelae:*** learning disabilities, neurological damage including epilepsy and hearing loss	<u>Costs</u>: Rehabilitation costs, drug costs, educational and social care costs<br><u>Outcomes</u>: Lifelong rehabilitation costs	<u>Patient A</u><br>*Total <u>medical costs</u>, discounted (undiscounted)*<br>12 months: $261,993<br>Lifetime costs: $951,294 ($1,797,614)

	documents by the national authorities)<br>Patient A: septicemia at 12 months of age; severe septic shock, acute respiratory distress syndrome and renal failure<br>Patient B: meningitis at 3 years of age; increased intracranial pressure, reduced conscious level and seizures; acute hydrocephalus requiring a shunt<br>Provider perspective<br><br>Time horizon: 12 months and lifetime (70 years)	***Psychological sequelae:*** behavior problems		*Total educational costs, discounted (undiscounted)*<br>Lifetime costs: $47,076 ($61,434)<br>*Total social care costs, discounted (undiscounted)*<br>12 months: $44,626<br>Lifetime costs: $843,744 ($1,707,616)<br><br>*Lifetime costs, discounted (undiscounted)*<br>Acute costs: $216,814 ($216,814)<br>Outpatient care: $14,170 ($20,861)<br>Prosthetic provision: $652,225 ($1,454,339)<br>Stump revisions and skin graft surgery: $25,310 ($33,756)<br>Psychological problems: $36,283 ($62,154)<br>Total lifetime costs: $1,842,115 ($3,566,633)<br><br>Patient B<br>*Total medical costs, discounted (undiscounted)*<br>12 months: $189,767<br>Lifetime costs: $532,076 ($1,095,405)<br>*Total educational costs, discounted (undiscounted)*<br>Lifetime costs: $240,771 ($314,437)<br>*Total social care costs, discounted (undiscounted)*<br>12 months: $51,888

				Lifetime costs: $1,431,364 ($3,493,566)<br><br>*Lifetime costs, discounted (undiscounted)*<br>Acute costs: $175,669 ($175,669)<br>Outpatient care: $28,435 ($67,138)<br>Cochlear implantation: $14,349 ($18,238)<br>Epilepsy: $99,610 ($288,721)<br>Shunt revision surgery: $12,689 ($22,111)<br>Total lifetime costs: $2,204,209 ($4,903,409)
Wright 2013[5]<br><br>UK	Two hypothetical cases (based on interviews, case studies, English Department of Health reference costs, British National Formulary, NHS catalogue of prosthetic components at the NHS supply chain)<br>Study period 2010–2011<br>Patient A: septicemia at 12 months of age; severe septic shock, acute respiratory distress syndrome and renal failure; gangrene of the limbs due to purpura fulminans<br>Patient B: meningitis at 3 years of age; raised intracranial pressure	Septicemia with sequelae:<br>***Physical:*** renal failure, multiple limb amputations, skin grafts,<br>***Psychological:*** Behavioral problems<br>Meningitis with sequelae:<br>***Physical:*** wheelchair confined, posture problems<br>***Neurological:*** epilepsy, cognitive deficits, hemiplegia, homonymous hemianopsia, deafness, communication problems	Costs: Hospital stay, inpatient, outpatient and acute care, health and social services, specialized equipment, medication, prosthetics, cochlear implants, revisions of stumps and skin graft surgery, psychological therapy, public health care, education, personal social services, other governmental costs, epilepsy management<br>Outcome: Lifetime government costs	Patient A<br>*Total cost, 12 months undiscounted*: $571,271<br>*Lifetime costs, discounted (undiscounted)*<br>Acute costs: $274,454 ($274,454)<br>Outpatient care: $56,936 ($99,842)<br>Prosthetic provision: $452,974 ($1,143,901)<br>Stump revisions and skin graft surgery: $38,072 ($53,823)<br>Behavioral problems: $14,643 ($19,638)<br>Education: $8,118 ($11,051)<br>Social care: $4,175 ($14,304)<br>Total cost: $3,426,176 ($7,497,117)<br><br>Patient B

	and intractable seizures, required ventilation and intubation; acute hydrocephalus<br>NHS and government perspective<br><br>Time horizon: 12 months and lifetime (70 years)			*Total cost, 12 months undiscounted*: $701,095<br>*Lifetime costs, discounted (undiscounted)*<br>Acute costs: $238,291 ($238,291)<br>Outpatient care: $35,335 ($61,295)<br>Cochlear implantation: $306,564 ($632,230)<br>Epilepsy: $6,532 ($16,623)<br>Shunt revision surgery: $32,511 ($58,121)<br>Education: $9,360 ($12,698)<br>Social care: $28,368 ($42,803)<br>Total cost: $4,907,644($13,621,059)
Wang 2019[6]<br><br>Australia	Model of 419 IMD cases (study period 2000)<br><br>N=419 IMD cases, adults aged 20 years and 30 years<br><br>Based on Published studies, National Hospital Cost Data Collection reports, Australian Refined Diagnosis Related Groups, expert opinion<br><br>Provider and societal perspectives<br><br>Time horizon: lifetime	***Physical sequelae:*** digit/limb amputations, arthritis, chronic renal failure, skin necrosis and grafting<br>***Neurological sequelae:*** Hearing loss, blindness, epilepsy, and brain injuries, severe speech and communication problems, chronic migraine<br>***Psychological sequelae:*** generalized anxiety disorder, depression	Costs: Direct healthcare costs of amputations, stump revisions and skin scars, direct non-healthcare/government subsidies costs associated with long-term care, informal carers, early intervention/special education, home/vehicle modification, and/or personal out of pocket costs<br>Outcomes: Productivity loss associated with acute admissions for patients with sequelae, average length of hospital stay, lifetime income foregone due to premature death	*Direct healthcare cost per IMD case by sequelae type, discounted*<br>Clinical follow-up cost for patients with disabilities requiring long term care: $12,059<br>Average long-term healthcare cost: $12,661<br>*Direct healthcare cost per IMD case by sequelae type*<br>Arthritis: $6,695<br>Blindness: $44,203<br>Brain injuries: $25,402<br>Chronic migraine: $11,038<br>Chronic renal failure: $281,316<br>Depression: $15,201<br>Digital amputation: $36,805<br>Epilepsy: $80,858<br>Generalized anxiety disorder: $11,038

				Hearing loss requiring adaptation strategies: $6,435<br>Hearing loss requiring cochlear implant: $21,179<br>Hearing loss requiring hearing aid: $15,920<br>Multiple limb amputation: $253,519<br>Severe speech problems: $44,203<br>Single limb amputation: $141,937<br>Skin grafting: $8,969<br>*Lifetime costs per IMD case*<br>Provider, 5% discount (healthcare): $14,593<br>Societal costs adopting friction cost, 5% discount: $25,188<br>Societal costs adopting human capital approach, 5% discount: $56,707
Wang 2014[7]<br><br>Australia	Cohort study (2000–2011)<br>N=109 IMD cases<br>Ages <one year<br>≥one year<br><br>Provider perspective<br><br>Time horizon: 1 year	***Physical sequelae:*** arthritis, knee pain, limb deformities, amputation, skin necrosis/ scarring, anemia and thrombocytosis, vasculitis,<br>***Neurological sequelae:*** Radiculopathy, subdural empyema, bilateral cranial nerve VI palsy, right cranial nerve VII palsy and optic disc swelling, hydrocephalus, multi cerebral infarct, visual impairment and developmental delay,	Costs: Medical ward, pathology, imaging, allied health (e.g. physiotherapy and speech pathology), pharmacy, use of theatre suite, the pediatric intensive care unit, prosthesis, medical and surgical supplies, hotel services, direct goods and services and overheads, inpatient costs during the IMD related readmissions<br>Outcomes: length of stay in hospital during the acute admissions for all patients and during IMD related readmissions for patients with sequelae. Number of IMD	*Inpatient costs per patient during acute hospitalization, unadjusted (adjusted)*<br>Mean number of inpatient days: 16.5 (14.3)<br>Cost: $34,983 ($29,521)<br><br>*Inpatient costs per patient during IMD related readmissions, unadjusted (adjusted)*<br>Mean number of inpatient days: 4.8 (1.1)<br>Cost: $9,490 ($1,122)

		esotropia, articulation problem, delayed speech and language skills, developmental delay, seizures/epilepsy, hearing impairment, chronic headaches, lethargy, gross motor difficulties, ***Psychological sequelae:*** Behavioral problems, autism, post-traumatic stress disorder	related outpatient visits and frequency of IMD related readmissions in patients with sequelae following the primary admission (incidence rate ratios -IRR). Inpatient costs during the readmissions for patients with sequelae by serogroup, age, gender, diagnosis type and absence or presence of a previous medical diagnosis	
Karve 2011[8]<br><br>US	Cohort study (1997–2009)<br>N=343 IMD patients<br>Infants, children, adolescents and adults<br>Ages 0–4 years, 5–10 years,<br>11–18 years, ≥19 years<br><br>Payer, insurance claims perspective<br><br>**Time horizon: 1 year**	***Physical sequelae:*** Adrenal hemorrhage, skin necrosis/skin grafting, limb amputation, chronic renal failure<br>***Neurological sequelae:*** hearing loss, blindness, seizure, epilepsy, learning disabilities, mental retardation, ataxia, hemiparesis, cranial nerve palsy, quadriparesis, obstructive hydrocephalus, anoxic brain damage and stroke	<u>Costs</u>: Inpatient, emergency department, outpatient, physician office, nursing home, and rehabilitation services, pharmacy utilization and associated costs<br><u>Outcomes</u>: Overall health care utilization and associated costs for the 12-month post index period. Health care utilization: a) percentage of patients with at least one claim for the selected category of service (e.g. inpatient, emergency department, physician office, pharmacy, hospital outpatient, home health/durable medical equipment, laboratory, and ancillary care); b) number of unique inpatient and emergency department admissions; c) length of hospital stay; d) number of physician office, outpatient, and other ancillary	Mean number of inpatient days: 25.6<br>Mean number of hospital outpatient visits: 12.7<br><br><u>Costs, unadjusted (adjusted by co-variates)</u><br>Mean total inpatient costs: $94,561 ($90,478)<br>Mean total emergency department costs: $1,550 ($1,531)<br>Mean total office visits costs: $3,507 ($4,071)<br>Mean total pharmacy costs: $2,237 ($3,454)<br>Mean total hospital outpatient costs: $16,700 ($14,209)<br>Mean total home health/durable medical equipment costs: $4,149 ($5,364)<br>Mean total laboratory services costs: $712 ($728)<br>Mean total ancillary care costs: $2,544 ($2,559)

			care visits and pharmacy claims; and e) percentage of patients with re-hospitalization during the post index period	Mean total costs: $125,961 ($122,227)

IMD, invasive meningococcal disease; N, Number; NHI, National Health Insurance; NHS, National Health Service; SNDS, Système National des Données de Santé (French National Healthcare Data System); UK, United Kingdom; US, United States; Y, Year

Table S3. Quality assessment CHEERS 2022 checklist[9]

Ref	Title	Abstract	Introduction	Methods																		Results				Discussion	Others	
	Q1	Q2	Q3	Q4	Q5	Q6	Q7	Q8	Q9	Q10	Q11	Q12	Q13	Q14	Q15	Q16	Q17	Q18	Q19	Q20	Q21	Q22	Q23	Q24	Q25	Q26	Q27	Q28
Karve 2011[8]	X	X	X	X	X	X	X	X	X	NA	X	X	X	X	X	X	X	X	X	X	NR	X	X	NR	NR	X	X	X
Wright 2013[5]	X	X	X	NR	X	X	X	X	X	X	X	X	X	X	X	NA	X	X	X	X	X	X	X	X	NR	X	X	X
Darba 2014[4]	X	X	X	NR	X	X	X	X	X	X	X	X	X	X	X	NA	X	X	X	X	X	X	X	X	NR	X	X	NR
Wang 2014[7]	X	X	X	NR	X	X	X	X	X	X	X	X	X	X	X	X	X	X	X	NR	NR	X	X	NR	NR	X	X	X
Benard 2016[2]	X	X	X	NR	X	X	X	X	X	X	X	X	X	X	X	NA	X	X	X	X	NR	X	X	X	NR	X	X	X
Scholz 2019[3]	X	X	X	NR	X	X	X	X	X	X	X	X	X	X	X	X	X	X	X	X	NA	X	X	X	NR	X	X	X
Wang 2019[6]	X	X	X	NR	X	X	X	X	X	X	X	X	X	X	X	X	X	X	X	X	NA	X	X	X	NR	X	X	X
Weil-Olivier 2021[1]	X	X	X	NR	X	X	X	X	X	NA	X	X	X	X	X	NA	X	X	X	NR	NR	X	X	NR	NR	X	X	X

CHEERS, Consolidated Health Economic Evaluation Reporting Standards; NA= Not applicable; NR= Not reported

Figure S1. Number and category of long-term IMD sequelae reported in studies

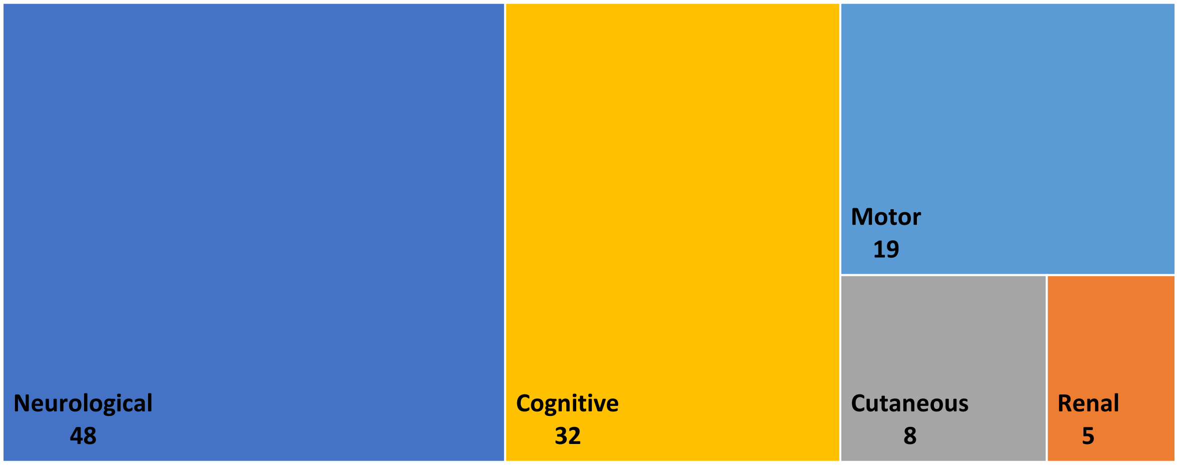

IMD, invasive meningococcal disease

First and second round of the Delphi survey

What is the average number of health resources used per patient with IMD-induced long-term sequelae in Brazil for the following?

Table S4. Amputation (1 limb)

Parameters	1st round			2nd round	
	Average	Range values (Min. – Max.)	Number of responders	Number of respondents agreeing	Number of respondents not agreeing
Amputation (1 limb) - first year					
Number of physiotherapy sessions/month	11	6-15	3	4	0
Number of visits to the emergency room/month	1.7	1-2	3	4	0
Total number of consultations with doctors/month	2.7	2-3	3	4	0
List specialists: Pediatrician or medical clinic, orthopedists, neurologist, infectious disease, psychiatrist, pain specialist, vascular	NA	NA	3	4	0
Number of therapy sessions with a psychologist/month	6.7	4-8	3	4	0
Number of hospitalization days/month	2	1-3	3	4	0
Purchase of provisional orthosis	1	NA	1	4	0
Adaptations at home	1	NA	1	4	0
Purchase of permanent orthosis	1	NA	1	4	0
Amputation (1 limb) - from 12 months					
Number of physiotherapy sessions/month	4.7	2-8	3	4	0
Number of visits to the emergency room/month	0.7*	0.2-1	3	4	0
Total number of consultations with doctors/month	1.3	1-2	3	4	0
List specialists: Pediatrician or medical clinic, orthopedists, neurologist, infectious disease, psychiatrist, pain specialist, vascular	NA	NA	3	4	0
Number of therapy sessions with a psychologist/month	4.7	2-8	3	4	0
Number of hospitalization days/month	0.5	0.5-1	3	4	0
Orthosis adjustments/year	2	NA	1	4	0
*One answer (0.2) not computed; ** All participants answered "1"					
Indirect costs associated with treating IMD-induced long-term sequelae					
Needs paid caregiver (In the first year)	Yes	NA	2	4	0
Needs paid caregiver (≥ 12 months)	No	NA	2	4	0
AVERAGE number of workdays missed by the family member (In the 1st year)	43	20-60	3	4	0
AVERAGE number of lost workdays/YEAR per household member (≥ 12 months)	14	10-20	3	4	0

Table S5. Amputation (2+ limbs)

Parameters	1st round			2nd round	
	Average	Range values (Min. – Max.)	Number of responders	Number of respondents agreeing	Number of respondents not agreeing
**Amputation (2 or more limbs) - first year**					
Number of physiotherapy sessions/month	12.4	4-20	5	5	0
Number of visits to the emergency room/month	2	1-4	5	5	0
Total number of consultations with doctors/month	2.4	1-3	5	5	0
List specialists:<br>Pediatrician or medical clinic, orthopedists, neurologist, infectious disease, psychiatrist, pain specialist, vascular	NA	NA	5	5	0
Number of therapy sessions with a psychologist/month	6	4-8	4	5	0
Number of hospitalization days/month**	2.3	2-3	3	5	0
Occupational therapy	8	NA	1	5	0
Speech therapist	8	NA	1	5	0
Prosthetist specialist	1	NA	1	5	0
Purchase of provisional orthosis	1	NA	1	5	0
Purch<br><br>ase of permanent orthosis	1	NA	1	5	0
Adaptations at home	1	NA	1	5	0
* Participants provided aggregated values for all specialists; ** One participant reported 4 months hospitalization at intensive care unit, outlier value not computed					
**Amputation (2 or more limbs) - from 12 months**					
Number of physiotherapy sessions/month	5.8	1-12	5	4	1*
Number of visits to the emergency room/month	0.6	0-1	4	5	0
Total number of consultations with doctors/month	1.2	1-2	5	5	0
List specialists:<br>Pediatrician or medical clinic, orthopedists, neurologist, infectious disease, psychiatrist, pain specialist, vascular	NA	NA	5	5	0
Number of therapy sessions with a psychologist/month	3.5	2-8	3	5	0
Number of hospitalization days/month	0.5	0.5-1	3	5	0
Occupational therapy	8	NA	1	5	0
Speech therapist	8	NA	1	5	0
Prosthetist	1	NA	1	5	0
Orthotic adjustments/year	2	NA	1	5	0
* Participant reported the average value=8					
**Indirect costs associated with treating IMD-induced long-term sequelae**					
Needs paid caregiver (In the first year)	Yes	NA	4	5	0
Needs paid caregiver (≥ 12 months)	Yes	NA	3	5	0
AVERAGE number of workdays missed by the family member (In the 1st year)	85	40-120	3	5	0
AVERAGE number of lost workdays/year per household member (≥ 12 months)	28	20-40	3	4	1*

* The participant reported that the average value is related to the first year only. The new value was not reported

Table S6. Skin scars

Parameters	1st round			2nd round	
	Average	Range values (Min. – Max.)	Number of responders	Number of respondents agreeing	Number of respondents not agreeing
Skin scars - first year					
Total number of outpatient visits for curative/month	2.7	2-4	3	5	0
Number of visits to the emergency room/month	1.3	1-2	3	5	0
Total number of consultations with doctors/month	1.5	1-2	3	5	0
List specialists<br>Pediatrician or medical clinic, dermatologist, neurologist, orthopedists, plastic surgeon, infectious disease, vascular	NA	NA	3	5	0
Number of therapy sessions with a psychologist/month	4.3	2-8	3	5	0
Number of hospitalization days/month	2	NA*	2	5	0
* Participants reported the same value	1st round			2nd round	
Skin scars - from 12 months					
Total number of outpatient visits for curative/month*	1.2	0.5-2	2	5	0
Number of visits to the emergency room/month**	0.4	0.2-1	2	5	0
Total number of consultations with doctors/month***	0.8	0.5-2	1	5	0
List specialties:<br>Pediatrician or medical clinic, dermatologist, neurologist, orthopedists, plastic surgeon, infectious disease, vascular	NA	NA	3	5	0
Number of therapy sessions with a psychologist/month	2.3	1-4	3	5	0
Number of hospitalization days/month****	0.7	0.3-1	2	5	0
* One answer not computed (0.5) not computed; ** One answer not computed (0.2) not computed; *** One answer not computed (0.5) not computed; **** One answer not computed (0.3) not computed; ***** Both participants reported 1					
Indirect costs associated with treating IMD-induced long-term sequelae					
Needs paid caregiver (In the first year)	No	NA	2	5	0
Needs paid caregiver (≥ 12 months)	No	NA	2	5	0
AVERAGE number of workdays missed by the family member (In the 1st year)	33	20-48	3	5	0
AVERAGE number of lost workdays/year per household member (≥ 12 months)	9	5-12	3	5	0

Table S7. Hearing loss/deafness

Parameters	1st round			2nd round	
	Average	Range values (Min. – Max.)	Number of responders	Number of respondents agreeing	Number of respondents not agreeing
**Hearing loss or deafness - first year**					
Number of visits to the emergency room/month*	1.1	0.5-2	3	4	0
Total number of consultations with doctors/month	2	1-4	4	4	0
List specialties<br>Pediatrician, neurologist, otorhinolaryngologist, medical clinic, speech therapist	NA	NA	4	4	0
Number of therapy sessions with a psychologist/month	4.7	2-8	3	4	0
Number of sessions with speech therapy/month	6	2-12	1	4	0
Hearing aid	NA	NA	1	4	0
* One answer not computed (0.5) not computed					
**Hearing loss or deafness - from 12 months**					
Number of visits to the emergency room/month*	0.4	0.2-1	2	4	0
Total number of consultations with doctors/month**	0.8	0.5-1	2	4	0
List specialties<br>pediatrician, neurologist, otorhinolaryngologist, medical clinic, speech therapist, psychiatrist	NA	NA	3	4	0
Number of therapy sessions with a psychologist/month	3	1-4	3	4	0
Number of sessions with speech therapy/month	3	1-4	3	4	0
* One answer not computed (0.2) not computed; **One answer not computed (0.5) not computed, both participants answered 1					
**Indirect costs associated with treating IMD-induced long-term sequelae**					
Needs paid caregiver (In the first year)	Yes	NA	2	4	0
Needs paid caregiver (≥ 12 months)	No	NA	2	4	0
AVERAGE number of workdays missed by the family member (In the 1st year)	39	30-48	3	4	0
AVERAGE number of lost workdays/year per household member (≥ 12 months)	16	5-24	3	4	0

Table S8. Epilepsy/seizures

Parameters	1st round			2nd round	
	Average	Range values (Min. – Max.)	Number of responders	Number of respondents agreeing	Number of respondents not agreeing
Epilepsy/seizures - first year					
Number of visits to the emergency room/month	1.8	1-2	4	4	0
Total number of consultations with doctors/month	2.5	1-4	4	4	0
List specialties<br>pediatrician, neurologist, medical clinic	NA	NA	4	4	0
Number of therapy sessions with a psychologist/month	4	2-8	3	4	0
Chronic use anticonvulsants	NA	NA	1	4	0
Epilepsy/seizures - from 12 months					
Number of visits to the emergency room/month*	0.8	0.3-1	2	4	0
Total number of consultations with doctors/month**	0.8	0.5-1	2	4	0
List specialties<br>Pediatrician, neurologist, medical clinic	NA	NA	3	4	0
Number of therapy sessions with a psychologist/month	2	1-4	3	4	0
Chronic use anticonvulsants	NR	NR	1	4	0
* One answer not computed (0.3) not computed, both participants answered 1; **One answer not computed (0.5) not computed, both participants answered 1					
Indirect costs associated with treating IMD-induced long-term sequelae					
Needs paid caregiver (In the first year)	Yes	NA	2	4	0
Needs paid caregiver (≥ 12 months)	Yes	NA	2	4	0
AVERAGE number of workdays missed by the family member (In the 1st year)	43	20-60	3	4	0
AVERAGE number of lost workdays/year per household member (≥ 12 months)	18	10-20	3	4	0

Table S9. Mental retardation/ low IQ

Parameters	1st round			2nd round	
	Average	Range values (Min. – Max.)	Number of responders	Number of respondents agreeing	Number of respondents not agreeing
Mental retardation/low IQ – first year					
Number of visits to the emergency room/month	3	1-4	3	4	0
Total number of consultations with doctors/month	3	2-4	3	4	0
List specialties:<br>Pediatrician, neurologist, medical clinic, psychiatrist	NA	NA	3	4	0
Number of therapy sessions with a psychologist/month	8.7	3-15	3	4	0
Occupational therapy	3	NA	1	4	0
Speech therapist	3	NA	1	4	0

Caregiver at home	NA	NA	1	4	0
Mental retardation/low IQ - from 12 months					
Number of visits to the emergency room/month	1.1	0.3-1	2	4	0
Total number of consultations with doctors/month	1	NA*	3	4	0
List specialists:<br>Pediatrician, neurologist, medical clinic, psychiatrist	NA	NA	3	4	0
Number of therapy sessions with a psychologist/month	3.3	2-4	3	4	0
Occupational therapy	1	NA	1	4	0
Speech therapist	1	NA	1	4	0
Caregiver at home	NA1	NA	1	4	0
* One answer not computed (0.3) not computed; ** All participants reported 1					
Indirect costs associated with treating IMD-induced long-term sequelae					
Needs paid caregiver (In the first year)	Yes	NA	3	4	0
Needs paid caregiver (≥ 12 months)	Yes	NA	3	4	0
AVERAGE number of workdays missed by the family member (In the 1st year)	59	20-96	3	4	0
AVERAGE number of lost workdays/year per household member (≥ 12 months)	21	10-30	3	4	0

Table S10. Mental health disorder

Parameters	1st round			2nd round	
	Average	Range values (Min. – Max.)	Number of responders	Number of respondents agreeing	Number of respondents not agreeing
Mental Health Disorder/anxiety/depression - first year					
Number of visits to the emergency room/month	2	1-4	3	4	0
Total number of consultations with doctors/month	3.3	2-4	3	4	0
List specialists<br>Pediatrician, neurologist, medical clinic, psychiatrist	NA	NA	3	4	0
Number of therapy sessions with a psychologist/month	9.7	6-15	3	4	0
Medications in general	NA	NA	1	4	0
Mental Health Disorder/anxiety/depression - from 12 months	1st round			2nd round	
Number of visits to the emergency room/month*	0.8	0.3-1	2	4	0
Total number of consultations with doctors/month	1.7	1-2	3	4	0
List specialists<br>Pediatrician, neurologist, medical clinic, psychiatrist	NA	NA	3	4	0
Number of therapy sessions with a psychologist/month	4	NA*	3	4	0
Medications in general	NA	NA	1	4	0

* One answer not computed (0.3) not computed, all participants reported 1; ** All participants reported 4		1st round			2nd round
Indirect costs associated with treating IMD-induced long-term sequelae	Average	Range values (Min. – Max.)	Number of responders	Number of respondents agreeing	Number of respondents not agreeing
Needs paid caregiver (In the first year)	Yes	NA	3	4	0
Needs paid caregiver (≥ 12 months)	Yes	NA	3	4	0
AVERAGE number of workdays missed by the family member (In the 1st year)	52	30-96	3	4	0
AVERAGE number of lost workdays/year per household member (≥ 12 months)	15	5-24	3	4	0

REFERENCES

1. Weil-Olivier C, Taha MK, Emery C, et al. Healthcare resource consumption and cost of invasive meningococcal disease in France: A study of the national health insurance database. Infectious diseases and therapy. 2021; 10: 1607-23. https://doi.org/10.1007/s40121-021-00468-w.

2. Bénard S, Wright C, Voisine J, et al. Lifetime cost of meningococcal disease in France: Scenarios of severe meningitis and septicemia with purpura fulminans. Journal of infection and public health. 2016; 9: 339-47. https://doi.org/10.1016/j.jiph.2015.10.016.

3. Scholz S, Koerber F, Meszaros K, et al. The cost-of-illness for invasive meningococcal disease caused by serogroup B Neisseria meningitidis (MenB) in Germany. Vaccine. 2019; 37: 1692-701. https://doi.org/10.1016/j.vaccine.2019.01.013.

4. Darbà J, Kaskens L, Hark M, et al. Costs of surviving meningococcal disease in Spain: evaluation for two cases of severe meningitis and septicaemia. Vaccine. 2014; 32: 5006-12. https://doi.org/10.1016/j.vaccine.2014.07.019.

5. Wright C, Wordsworth R, Glennie L. Counting the cost of meningococcal disease : scenarios of severe meningitis and septicemia. Paediatric drugs. 2013; 15: 49-58. https://doi.org/10.1007/s40272-012-0006-0.

6. Wang B, Haji Ali Afzali H, Giles L, et al. Lifetime costs of invasive meningococcal disease: A Markov model approach. Vaccine. 2019; 37: 6885-93. https://doi.org/10.1016/j.vaccine.2019.09.060.

7. Wang B, Haji Ali Afzali H, Marshall H. The inpatient costs and hospital service use associated with invasive meningococcal disease in South Australian children. Vaccine. 2014; 32: 4791-8. https://doi.org/10.1016/j.vaccine.2014.05.069.

8. Karve S, Misurski D, Miller J, et al. Costs of sequelae associated with invasive meningococcal disease: Findings from a US managed care population. Health Outcomes Research in Medicine. 2011; 2: e215-e26. https://doi.org/10.1016/j.ehrm.2011.08.001.

9. Husereau D, Drummond M, Augustovski F, et al. Consolidated Health Economic Evaluation Reporting Standards (CHEERS) 2022 Explanation and Elaboration: A Report of the ISPOR CHEERS II Good Practices Task Force. Value in health : the journal of the International Society for Pharmacoeconomics and Outcomes Research. 2022; 25: 10-31. https://doi.org/10.1016/j.jval.2021.10.008.
